# ER-Negative Breast Cancer Is Highly Responsive to Cholesterol Metabolite Signalling

**DOI:** 10.3390/nu11112618

**Published:** 2019-11-01

**Authors:** Samantha A Hutchinson, Priscilia Lianto, Hanne Roberg-Larsen, Sebastiano Battaglia, Thomas A Hughes, James L Thorne

**Affiliations:** 1 School of Food Science and Nutrition, University of Leeds, Leeds LS2 9JT, UK; 2 Department of Chemistry, University of Oslo, 0315 Oslo, Norway; 3 Center for Immunotherapy, Roswell Park Cancer Institute, Buffalo, 14263 NY, USA; 4 School of Medicine, University of Leeds, Leeds LS9 7TF, UK; t.hughes@leeds.ac.uk

**Keywords:** cholesterol, hydroxycholesterol, breast cancer, LXR, oestrogen receptor status, corepressors

## Abstract

Interventions that alter cholesterol have differential impacts on hormone receptor positive- and negative-breast cancer risk and prognosis. This implies differential regulation or response to cholesterol within different breast cancer subtypes. We evaluated differences in side-chain hydroxycholesterol and liver X nuclear receptor signalling between Oestrogen Receptor (ER)-positive and ER-negative breast cancers and cell lines. Cell line models of ER-positive and ER-negative disease were treated with Liver X Receptor (LXR) ligands and transcriptional activity assessed using luciferase reporters, qPCR and MTT. Publicly available datasets were mined to identify differences between ER-negative and ER-positive tumours and siRNA was used to suppress candidate regulators. Compared to ER-positive breast cancer, ER-negative breast cancer cells were highly responsive to LXR agonists. In primary disease and cell lines LXRA expression was strongly correlated with its target genes in ER-negative but not ER-positive disease. Expression of LXR’s corepressors (NCOR1, NCOR2 and LCOR) was significantly higher in ER-positive disease relative to ER-negative, and their knock-down equalized sensitivity to ligand between subtypes in reporter, gene expression and viability assays. Our data support further evaluation of dietary and pharmacological targeting of cholesterol metabolism as an adjunct to existing therapies for ER-negative and ER-positive breast cancer patients.

## 1. Introduction

Cholesterol is predominantly synthesized de novo in the liver with lesser amounts obtained from the diet. Dietary intake, de novo synthesis, metabolism and excretion, combine to balance circulating cholesterol levels ensuring extra-hepatic tissues are sufficiently equipped to produce a range of metabolites including steroid hormones, bile acids and seco-steroids. Side-chain hydroxycholesterols (scOHCs) are typically formed through hydroxylation of cholesterol by specialized members of the Cytochrome P450 family, which bind and activate the Liver X Receptor-alpha (LXRA; gene name *NR1H3*) and beta (LXRB; gene name *NR1H2*) transcription factors [1,2]. LXR target genes are typically involved in cholesterol and fatty acid metabolism. In normal tissue, expression of LXRA is inducible in the liver, intestine, macrophages and adipocytes, whilst expression of LXRB is more ubiquitous. As well as differences in expression of LXRA and LXRB, local concentrations of the scOHCs differ considerably between tissues, and relative to each other, sometimes by as much as 1000-fold [3] and variance can also depend on disease status [4]. Furthermore, the different scOHCs have varying capacities to activate LXR-mediated transcription, imposing an element of selective modulation onto signalling. 26-OHC (commonly referred to as 27-OHC [5]) for example is the most abundant scOHC, but is a relatively weak LXR agonist [1,6]. Moreover, there is little difference in scOHC concentrations between breast cancer subtypes [7].

Transcriptional activity of the LXRs, like the other members of the Nuclear Receptor (NR) superfamily, is not just regulated by ligand bioavailability; chromatin environment, cross-talk and competition for response element binding [8] with other NRs, as well as cell- and tissue-type dependent expression of cofactors are also key mediators. For example, the expression of corepressors such as NCOR1 and NCOR2/SMRT determine how several cancers respond to nutritive ligands [9,10,11]. LXRA has a 100-fold higher binding affinity than LXRB for the corepressors NCOR1 and NCOR2 [12] and deregulation of these corepressors allows prostate and bladder cancer cells to evade cancer suppressive signals of Vitamin D (through repression of Vitamin D Receptor (VDR)) and omega-3 fatty acids (through repression of peroxisome proliferator-activated receptors (PPARs)) by impairing sensitivity to ligand [10,11,13]. Simply measuring scOHC concentrations does not sufficiently determine their contributions to LXR signalling; concentration and activation potential should be assessed in combination.

In cancer, the function of the scOHC-LXR signalling axis appears site specific as both tumour suppressive and promoting roles have been described. For example, scOHC-LXR signalling impairs invasion and angiogenesis in melanoma [14] and is anti-proliferative in lung cancer in vivo [15], as it is in almost every cancer cell line studied in vitro [13]. In Oestrogen Receptor (ER)-positive Breast Cancer (BCa) however 26-OHC promotes growth in vivo via ER-alpha [4,16]. In ER-negative BCa 26-OHC drives the epithelial-to-mesenchymal transition [16] and promotes colonization of metastatic sites in through mobilization of γδ-T cells [17]. Furthermore, concentrations of several scOHCs are altered in BCa relative to normal tissue [4], and 25-OHC is elevated in the circulation of BCa patients who have relapsed compared to those with primary disease [18].

We recently evaluated LXR ligand bio-availability in a small BCa cohort [7] and found large inter tumoural heterogeneity in oxysterol content, but no difference in ligand concentrations between tumour subtypes. Systematic evaluation of scOHC bioavailability and activation potential, coupled with analysis of NR cofactor expression between BCa subtypes has not been performed previously. Given the prognostic and therapeutic value of stratifying BCa by hormone receptor status, further delineation of the pathways that are altered between these subtypes, such as scOHC-LXR signalling, may help advance understanding about the emerging roles of cholesterol metabolism in cancer and improve outcomes for patients.

## 2. Materials and Methods

### 2.1. Cell Culture and Transfections

MCF7, T47D, MDA-MB-468 and MDA-MB-231 cell lines were originally obtained from ATCC. All cells were maintained at 37 °C with 5% CO_2_ in a humidified incubator and cultured in Dulbecco’s Modified Eagle Medium (DMEM, Thermo Fisher, Altrincham, UK Cat: 31966047) supplemented with 10% fetal calf serum (FCS) (Thermo Fisher, UK, Cat: 11560636). Routine passaging of cells was completed every 3–4 days, and seeded at 1 × 10^6^ live cells per T75 tissue culture treated flask (Nunc, Thermo Fisher, UK, Cat: 10364131) to maintain confluence between 20% and 80%.

For transfection with siRNA, cells were plated in 6-well plates (MDA-MB-468 cells: 1.5 × 10^5^ cells/well; MCF7 cells: 1 × 10^5^ cells/well) and incubated overnight. Lipofectamine RNAiMAX (Thermo Fisher, Cat: 13778030), siNCOR1 (Cat: SR306392), siNCOR2 (Cat: SR306393) or siLCOR (Cat: SR313532), or the scrambled siRNA (Cat: SR30004) were diluted in OptiMeM (Thermo Fisher, Cat: 31985062), combined according to manufacturer’s instructions, and added to the cells at a final concentration of 30 nM. The cells were incubated for 20 h and the media was changed for fresh DMEM. Cells were plated for luciferase or qPCR assays after 36 h, and for MTT at 24 h, knockdown was confirmed at mRNA level at 36 h.

### 2.2. Drugs and Reagents

Drugs stocks were stored at −20 °C as follows: GSK2033 (gift from Dr Carolyn Cummings—University of Toronto, Toronto, ON, Canada) at 20 mM diluted in DMSO. GW3965 (Cayman, Ann Arbor, MI, USA, Cat: 71810) at 100 mM diluted in DMSO. Hydroxycholesterols were sourced from Avanti Polar Lipids (Alabaster, AL, USA): 7-ketocholesterol (7-KETO) (Cat: 700015), 22R-hydroxycholesterol (22-OHC) (Cat: 700058), 24S-hydroxycholesterol (24-OHC) (Cat: 700071), 25-hydroxycholesterol (25-OHC) (Cat: 700019), 26-hydroxycholesterol (26-OHC) (Cat: 700021) and 24(R/S),25-epoxycholesterol (24,25-EC) (Cat: 700037). Stocks of 10 mM were prepared in nitrogen flushed ethanol (NFE) to prevent auto-oxidation. Puromycin Hydrochloride (Santa Cruz, CA, USA; Dallas, TX, USA, Cat: sc-108071) stocks diluted in Nuclease Free Water and stored as 25 mg/mL aliquots.

### 2.3. Luciferase Reporter Assay

This method has been reported previously [19]. Briefly, 3 × 10^4^ cells were plated in each well of a 24-well plate and incubated overnight. Cignal Lentiviral particles (LXRA) were purchased from Qiagen, Manchester, UK (Cat: CLS-7041L) and transduced into the cells using 8 µg/mL SureEntry transduction reagent at MOI at manufacturer’s recommendations. After 18 h the particles were removed and fresh DMEM supplemented with 0.1 mM Non Essential Amino Acids (Thermo Fisher, Cat: 12084947) and 100 U/mL penicillin and 100 μg/mL streptomycin (Thermo Fisher, Cat: 10378016) were added to the cells. Cells were passaged and puromycin used to select successfully transduced cells. For luciferase quantitation, 30,000 transfected cells/well were seeded into 24-well plates, and allowed to attach under normal culture conditions for 8 h. Cultures were treated with ligands, inhibitors or vehicle control as indicated in figure legends for 16 h. Luciferase assays were carried out by transferring cell lysates to white-walled 96-well plates and luminescence was assessed using the Tecan Spark 10M.

### 2.4. qPCR

Cells were plated in 6-well plates (2.5 × 10^5^ cells/well) and incubated overnight before treatment with Vehicle Control (ETOH) or LXRA ligands. mRNA analysis was performed as described previously [20,21]. Briefly, Promega Reliaprep^TM^ RNA Cell Miniprep System was used for the RNA extraction (Promega, Southampton, UK, Cat: #Z6012), and product guidelines were followed using approximately 5 × 10^5^ cells. On column DNase 1 digestion was performed and RNA was eluted in 30 μL nuclease free water. RNA was stored at −80 °C. GoScript^TM^ Reverse Transcription kit (Promega, UK, Cat: A5003) was used for the cDNA synthesis, and product guidelines followed (TM316), using 500 ng total RNA/reaction and 0.5 μg/μL random primers. cDNA produced was then diluted 1 in 5 in nuclease free water and stored at −20 °C. For gene expression analysis, Taqman Fast Advanced Mastermix (Thermo Fisher, Paisley, UK, Cat: 4444557) was used with Taqman assays (Thermo Fisher, Paisley, UK, Cat: 4331182) on a QuantStudio Flex 7 (Applied Biosystems Life Tech, Thermo Fisher, Paisley, UK) in 384-well plates. Taqman assays and Mastermix were stored at −20 °C. Taqman ID’s used were *HPRT1*: Hs02800695_m1; *ABCA1*: Hs01059137_m1; *APOE*: Hs00171168_m1; *DOK2*: Hs00929587_m1; *LCP2*: Hs01092638_m1; *TNFRSF1B*: Hs00961750_m1; *LCOR*: Hs00287120_m1; *NCOR1*: Hs01094540_m1; *NCOR2*: Hs00196955_m1. Gene expression was analysed using the ΔΔcT method and normalised to *HPRT1*. *HPRT1* was confirmed as the most suitable housekeeping gene from a panel of 18 housekeeping genes tested in MCF7 and MDA-MB-468 cell lines treated with a panel of sterols at various time points and in various concentrations (Thermo Fisher, Paisley, UK, Cat: 4367563).

### 2.5. MTT Assays

The human BCa cell lines (MCF7, MDA-MB-468) were cultured in DMEM (glucose 4.5 g/L) supplemented with 10% fetal bovine serum (FBS) at 37 °C in a humidified 5% CO_2_ incubator. Seeding density was determined empirically for each cell line and for each time point. Then, 2 × 10^4^ cells/well for MDA-MB-468 cell line and 3 × 10^4^ cells/well for MCF7 were seeded in 96-well plates. After overnight incubation, media was removed and replaced with the fresh media (200 μL) with vehicle control (0.1% ethanol flushed with N_2_) or 10 μM, or 100 μM 26-OHC (in vehicle) for indicated time points. Cells were washed with PBS and 90 μL phenol-red free DMEM was added with 10 μL of diluted MTT reagent at 37 °C for 4 h incubation. Media was removed and 100 μL of DMSO was added, absorbance was read using a CLARIOstar plate reader at 540 nm.

### 2.6. The Cancer Genome Atlas Gene Expression Analysis

To establish the possible regulators of LXR activity, cofactors were included if they physically interacted with LXR in a previously performed NR/cofactor scan [22], and if the interaction had been reported in at least one other study. Based on these criteria, a total of six coactivators and three corepressors were selected for further analysis. mRNA expression of was assessed using the array-median centered gene expression obtained from http://cBioportal.org [23], deposited by The Cancer Genome Atlas (TCGA) BCa dataset [24]. Data collection and analysis was performed as described previously [25,26]. Expression data were obtained for 81 Basal (ER-/PR-/Her2-) and 234 Luminal A (ER+/PR+/Her2-) tumours and were compared using two-tailed Mann–Whitney U tests using Bonferroni correction for multiple testing where indicated [27].

### 2.7. Transcription Factor-Target Gene Correlation Analysis

NR1H3/LXRA binding to gene promoters was assessed in all available ChIP-Seq datasets deposited into the http://cistrome.org portal [28]. LXRA binding scores were obtained from seven ChIP-Seq datasets, from three publications that had deposited LXRA binding information for mouse monocytes either untreated or exposed to LXRA agonist GW3965 [29], human colorectal cancer cells treated with GW3965 at 2 or 48 h [30] (both time points had duplicate ChIP-Seq datasets associated with them, which were averaged to give a 2 h list and a 48 h list), and untreated human adipocytes [31]. Processed ChIP-Seq data were accessed and the 100 genes with the highest LXRA binding scores in each of the seven datasets were included for further analysis. If a gene was present in the top 100 bound genes in only one dataset it was excluded from further analysis (resulting in 148 genes appearing in multiple lists). Eleven genes (from mouse datasets) were excluded as they did not have human orthologs, and a further 26 were excluded as they were not expressed in the TCGA dataset resulting in a 111 gene list. To this list, 24 canonical LXR targets identified from the literature were included in analysis, even if they did not necessarily reach the cut-off criteria outlined above. The entire list of 135 genes was then assessed for Pearson correlation with NR1H3/LXRA in the 81 ER-negative and 234 ER-positive breast tumours from TCGA [24]. The Pearson’s correlation coefficient was calculated between each of the 135 genes and LXRA and the resulting *p* values were corrected for False Discovery Rate (FDR). Lastly, Fisher’s exact test was used by building a contingency table to test the null hypothesis that the number of genes with FDR of 1% is the same in the two diseases. Flow diagram of gene selection and exclusion methodology can be found in Supplementary Materials (Figure S1).

## 3. Results

### 3.1. LXR Activation Potential Is Retained in ER-Negative Disease but Dampened in ER-Positive Disease

**Figure 1:**
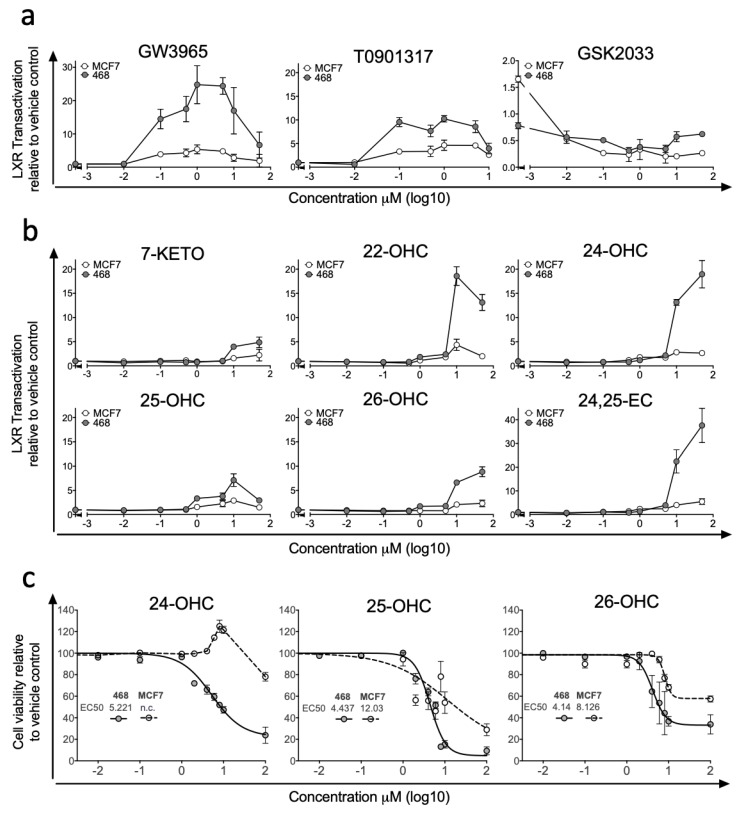
Synthetic ligands and side-chain hydroxycholesterols (scOHCs) activate Liver X Receptor-alpha (LXRA) dependent transcription in Oestrogen Receptor (ER)-negative but not ER-positive breast cancer cell culture. ER-negative (MDA-MB-468) and ER-positive (MCF7) cell lines were stably transfected with LXRA-Luciferase reporter constructs and treated with synthetic LXR agonists or the antagonist GSK2033 (**a**), or endogenous LXR ligands (**b**) at indicted concentrations. The anti-proliferative effects of scOHC over 48 h was assessed by MTT in MDA-MB-468 and MCF7 cells (**c**) with EC50 given in μM. Data are presented as means of 2–4 independent replicates with SEM.

Previous studies examining differences in LXR signalling across BCa ER-subtypes have reported strong anti-proliferative actions of synthetic LXR agonists in ER-positive cell lines, but enhanced transcription from canonical gene loci such as ABCA1 and SREBP1c in ER-negative cell lines [32]. To explore differences in how breast cancer cells respond to LXR stimulation by synthetic and endogenous ligands, we generated LXRA-regulated luciferase reporter cell lines representing ER-negative (MDA-MB-468, MDA-MB-231) and ER-positive (MCF7, T47D) BCa, as well as a control liver cell line (HEPG2). Dose-response experiments were performed with synthetic LXR ligands (agonists: T0901317 and GW3965 [33]; inhibitor: GSK2033 [34]). At nanomolar and micromolar concentrations in MDA-MB-468 cell culture, GW3965 treatment resulted in up to a 25-fold induction of LXR driven luciferase activity and T0901317 resulted in up to 10-fold induction (Figure 1a). When attempting to stimulate LXR-mediated transcription in the ER-positive MCF7 cell line activation was restricted to less than 5-fold above vehicle control for both synthetic agonists (Figure 1a). We repeated GW3965 treatment in additional ER-positive (T47D) and ER-negative (MDA-MB-231) lines confirming our observation that ER-negative cells were significantly more responsive to LXR agonist than ER-positive cells in LXR-reporter assay (one-tailed students *t*-test: *p* < 0.0001 (Figure S2a)). GSK2033 suppressed basal LXR dependent transactivation similarly in both MCF7 and MDA-MB-468 cell cultures (Figure 1a). We then applied a panel of endogenous LXR ligands (7-KETO, 22-OHC, 24-OHC, 25-OHC, 26-OHC and 24,25-EC) and found LXR was activated in both cell lines by all ligands but to varying amounts. Similarly to the synthetic ligands, activation was more robust in MDA-MB-468 compared to MCF7 cells, across all ligands and all concentrations tested (Figure 1b). In the MDA-MB-468 reporter cells, 24,25-EC induced the greatest fold change in LXR dependent luciferase expression (×40-fold increase), followed by 22-OHC (×19-fold) and 24-OHC (×18-fold). Induction by 26-OHC (×9-fold), 25-OHC (×6-fold) and 7-KETO (×5-fold) were relatively attenuated (Figure 1b). In contrast, the maximum induction by any scOHC observed in MCF7 cells was <5-fold. As control experiments we first generated a stable luciferase reporter liver cell line HEPG2 and activation by agonists and repression by antagonists in HEPG2 was comparable to that observed in MDA-MB-468 cells (Figure S2b). In the absence of LXRA (following siRNA knockdown) basal luciferase activity was lowered and neither 26-OHC (Figure S2c) or 24,25-OHC (Figure S2d) treatment elicited any induction of luciferase activity, demonstrating the dependence on LXRA. As scOHC-LXR signalling is known to be anti-proliferative and pro-apoptotic in an array of cell lines, we applied MTT assays to test whether ER-negative MDA-MB-468 cells were more sensitive to ligands than their ER-positive MCF7 counterparts in an alternative assay. MCF7 cells were significantly more resistant than MDA-MB-468 cells to treatment with 24-OHC (non-linear regression comparison of fits: non-converged for MCF7), 25-OHC (*p* < 0.0001) and 26-OHC (*p* < 0.0001) (Figure 1c).

**Figure 2:**
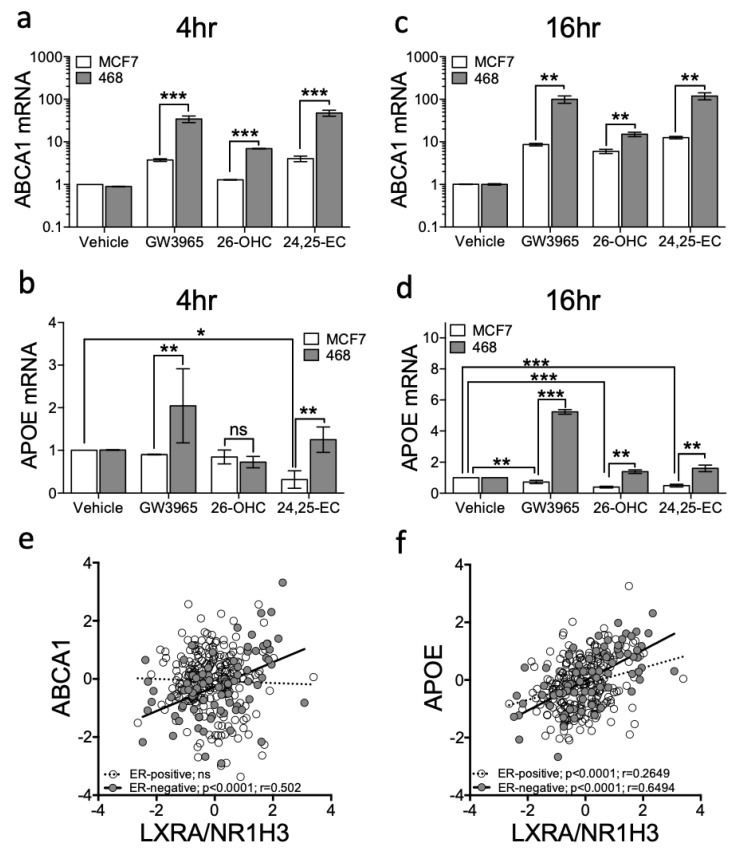
Ligand dependent transcriptional output of LXR target genes is enhanced in ER-negative relative to ER-positive breast cancer cell cultures. ER-negative (MDA-MB-468) and ER-positive (MCF7) cell lines were treated with a panel of ligands (Vehicle control, GW3965 (1 μM), 26-OHC or 24,25-EC (both 10 μM)) for 4 (**a**,**b**) and 16 h (**c**,**d**). Expression of the canonical LXR target genes *ABCA1* (**a**,**c**) and *APOE* (**b**,**d**) were assessed by qPCR using ΔΔcT (normalised to HPRT1). Statistical analysis was established using multiple *t*-tests and data are derived from three independent replicates with SEM. mRNA-Seq data from TGCA for 81 ER-negative and 234 Luminal A tumours was assessed using Pearson correlation between *NR1H3/LXRA* and *ABCA1* (**e**) or *APOE* (**f**). * *p* < 0.05, ** *p* < 0.01, *** *p* < 0.001. Lines represent linear regression.

To confirm the luciferase LXRA reporter was representative of regulation within a normal chromosomal context, we next examined expression at two endogenous canonical LXR target loci, *ABCA1* [35] and *APOE* [36]. Vehicle control, GW3965, 26-OHC (the most abundant scOHC in breast tumour tissue [7]) or 24,25-EC (the scOHC that elicited the greatest fold induction in reporter cells (Figure 1b)) were added to MDA-MB-468 or MCF7 cells for 4 or 16 h and changes to *ABCA1* and *APOE* expression determined. At 4 h *ABCA1* was induced in both cell lines by GW3965 and 24,25-EC but not by 26-OHC in MCF7 cells (Figure 2a). Induction was greater after treatment in MDA-MB-468 compared to MCF7 cells (multiple *t*-tests with FDR < 1% and Holm–Sidak correction: GW3965 *p* = 0.0097; 26-OHC *p* = 0.0092; 24,25-EC *p* = 0.0086; Figure 2a). GW3965, but not other agonists, induced *APOE* induction at 4 h in MDA-MB-468 cells (Figure 2b). At 16 h *ABCA1* was induced by all ligands in both cell lines, but again, to a significantly greater level in MDA-MB-468 cells (multiple *t*-tests with FDR < 1% and Holm–Sidak correction: GW3965 *p* < 0.01; 26-OHC *p* < 0.01; 24,25-EC *p* < 0.01; Figure 2c). At 16 h *APOE* was also induced by all ligands in MDA-MB-468 cells but interestingly, was repressed by 26-OHC and 24,25-EC (but not synthetic ligand) in MCF7 cells (compare columns 3 and 4 against vehicle; Figure 2d), and by 24,25-EC at 4 h (multiple *t*-tests with FDR < 1% and Holm–Sidak correction: *p* = 0.003). We repeated GW3965 treatment in second ER-positive (T47D) and ER-negative (MDA-MB-231) lines confirming our observations that *ABCA1* (two-way ANOVA: *p* < 0.05 (Figure S3a)) and *APOE* (two-way ANOVA: *p* < 0.001 (Figure S3b)), were significantly more induced in ER-negative cells compared to the ER-positive ones. Accumulation of *ABCA1* mRNA was LXR dependent, as the LXR inhibitor GSK2033 abrogated the scOHC response in both cell lines (Figure S4a) and knockdown of *LXRA* impaired *ABCA1* expression (Figure S4b). From these findings we concluded that at the transcriptional level, ER-negative cells are more responsive to LXR stimulation than their ER-positive counterparts.

We next set out to establish if the enhanced LXR transcriptional activity observed in cell line models extended to primary tumours. To test this, we examined whether expression of *NR1H3/LXRA* or *NR1H2/LXRB* correlated with expression of canonical LXR target genes (*ABCA1* and *APOE*) in 81 ER-negative or 234 ER-positive primary breast tumours (obtained from TCGA dataset [24]). *ABCA1* correlated with *LXRA* (Pearson’s correlation: R = 0.502: *p* < 0.0001) in ER-negative but not in ER-positive tumours (Figure 2e). *APOE* correlated with *LXRA* in both subtypes (Figure 2f), but the correlation was much weaker in ER-positive than in ER-negative disease (Pearson correlation: ER-positive: R = 0.27, *p* < 0.0001; ER-negative: R = 0.65: *p* < 0.0001). Both *ABCA1* and *APOE* were assessed for correlation with *NR1H2/LXRB* and whilst *APOE* weakly correlated with *NR1H2/LXRB* in ER-positive tumours (R = 0.25) it was not correlated in ER-negative tumours; *ABCA1* was not correlated with *NR1H2*/*LXRB* in either tumour type (Figure S5). From these observations we concluded that ER-status was inversely associated with the ability of LXR to induce canonical target gene expression.

### 3.2. Expression of LXRA Correlates with Expression of Its Target Genes in Primary ER-Negative Tumours but not in ER-Positive Tumours

**Figure 3:**
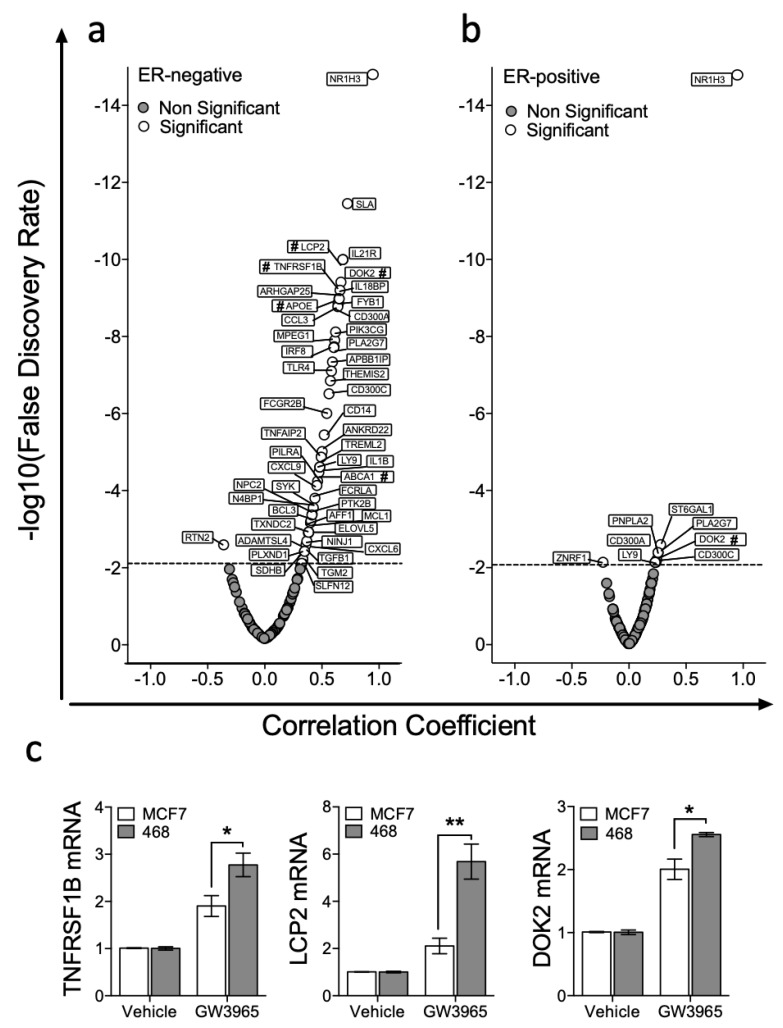
LXRA expression correlates with target genes in ER-negative tumours but not in ER-positive BCa. Genes with top LXRA occupancy scores from the seven NR1H3/LXRA ChIP-Seq datasets available at cistrome.org were identified along with 24 canonical LXR targets identified from the literature, and correlation with *LXRA/NR1H3* expression in 81 ER-negative and 234 Luminal A tumours from The Cancer Genome Atlas (TCGA) determined. Dotted line denotes false discovery rate corrected for multiple testing of expression of 135 genes. Data presented are correlation coefficients against correlation significance in ER-negative (**a**) and ER-positive (**b**) breast tumours. Genes marked with # were validated by qPCR analysis in (**c**) where ER-negative (MDA-MB-468) and ER-positive (MCF7) cell lines were treated with either Vehicle control or GW3965 (1 μM) and expression of three highly significant genes (TNFRSF1B, LCP2 and DOK2) determined. Statistical significance was tested for using multiple *t*-tests (corrected with Holm–Sidak) and shows three independent replicates with SEM. * *p* < 0.05, ** *p* < 0.01.

We then set out to test if expression of a wider and unbiased set of LXR target genes correlated with *NR1H3/LXRA* or *NR1H2/LXRB* expression in ER-positive or ER-negative tumours. A list of LXRA target genes was generated ‘agnostically’ by repeated interrogation of publicly available ChIP-Seq data sets as described above (full gene list in ST1 and example promoters shown in Figure S6 [29]) using cistrome.org [28]. Then, we assessed correlation of expression of each of these LXRA bound gene targets with *NR1H3/LXRA* and *NR1H2/LXRB* expression in publicly available RNA-Seq datasets from TCGA, as previously for *ABCA1* and *APOE*. In ER-negative tumours *NR1H3/LXRA* significantly correlated with 48/135 genes (Figure 3a), compared to 8/135 in ER-positive tumours (Figure 3b). This was a statistically significant difference in the number of correlating genes (Fisher’s exact test: *p* < 0.0001). Three genes that had not previously been validated as bona fide LXR target genes (*TNFRSF1B*, *LCP2* and *DOK2*) were selected from the top 10 strongest correlations, to test the in silico prediction that these genes should be inducible in MDA-MB-468 cells, but not (or less so) in MCF7 cells. qPCR analysis on cells exposed for 16 h to 1 μM GW3965 revealed that all three genes were induced to significantly greater extent in the MDA-MB-468 cell line than in MCF7 (multiple *t*-tests with FDR < 1% and correction for multiple testing with Holm–Sidak: *TNFRSF1B p* = 0.033; *LCP2 p* = 0.006; *DOK2 p* = 0.015) (Figure 3c). We concluded that retention of LXRA’s transcriptional potential was associated with more stringent correlations between LXRA and its target genes in vivo, and more robust activation of target genes in vitro.

### 3.3. LXR Is Poised for Transcription in ER-Negative BCa but Repressed in ER-Positive BCa

The capacity for NRs to regulate their target genes depends on multiple factors, including receptor expression, ligand bioavailability and coactivator/corepressor expression. As we previously found no difference in ligand concentrations [7], we hypothesized that the balance of functional regulators of LXR would be different between ER-positive and ER-negative BCa.

**Figure 4:**
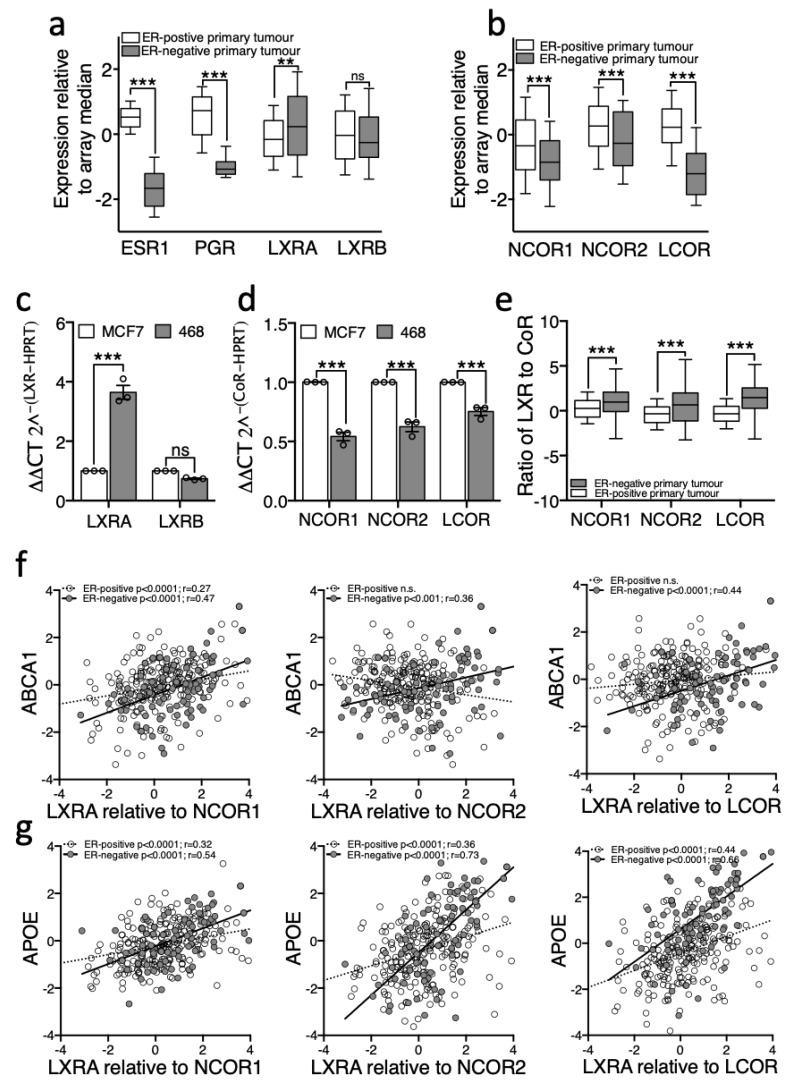
LXRA and its corepressors are differentially expressed between primary ER-negative and ER-positive breast cancers. RNA-Seq gene expression data (log transformed relative to array median) were obtained for 81 ER-negative and 234 Luminal A tumours from TCGA via cBioportal. NR (**a**) and CoR (**b**) expression was determined in from the TCGA database and in cell lines MDA-MB-468 and MCF7 (**c**,**d**). Expression of LXR relative to corepressor in the TCGA data is shown in (**e**). TCGA data are presented as log transformed and normalized to array-median with 10–90th centiles (a,c,e). Error bars represent SEM of 3–4 independent replicates for cell line analysis (**c**,**d**). Statistical analysis was performed using two-tailed Mann–Whitney U tests for (a,b), two-tailed student *t*-test (c,d), or Pearson’s correlation (**f**,**g**). ** *p* < 0.01, *** *p* < 0.001, ns = not significant.

Expression of relevant genes was assessed in 234 ER-positive and 81 ER-negative human tumours from TCGA [24]. First, as a control, we show that as expected oestrogen receptor alpha (*ESR1*) and progesterone receptor (*PGR*) were significantly different between these groups, with median expression in ER-negative tumours dramatically lower than in ER-positive tumours (Figure 4a). Next, more interestingly, *NR1H3/LXRA* was expressed at higher levels in ER-negative than in ER-positive tumours (two-tailed Mann–Whitney U test: *p* < 0.01 (Figure 4a)). *NR1H2/LXRB* was unchanged between subtypes. In the absence of agonist, LXRA but not LXRB, is primarily repressed by corepressors NCOR1 and NCOR2/SMRT [12] and previous reports demonstrate elevated corepressor expression helps prostate [9,10] and bladder [11] cancer cells to evade anti-proliferative actions of NRs through compromising the ligand response. Both NCOR1 and NCOR2/SMRT were expressed at significantly lower levels in ER-negative tumours compared to ER-positive tumours (two-tailed Mann–Whitney U test: *p* < 0.001 (Figure 4b)). Interestingly, expression of LCOR, a corepressor that is recruited to LXR upon agonist binding [37], was even more drastically reduced in primary ER-negative tumours (two-tailed Mann–Whitney U test: *p* < 0.0001, (Figure 4b)). We repeated these measurements in vitro and found the cell line models recapitulated these features of the primary tumours; MDA-MB-468 expressed significantly more LXRA but not LXRB (Figure 4c), and significantly less *NCOR1*, *NCOR2/SMRT* and *LCOR* transcript than the ER-positive cell line MCF7 (two-tailed student’s *t*-test: *p* < 0.0001 for all corepressors (Figure 4d)). In a reanalysis of a previously published expression dataset of BCa cell lines [38], we found that *NCOR2/SMRT* and *LCOR* (but not *NCOR1*) were also expressed at significantly lower levels in ER-negative cell lines generally compared to ER-positive cell lines (Mann–Whitney-U test: *p* < 0.05 for NCOR2 and LCOR (Figure S7)).

Next, we hypothesised that if corepressors were responsible for the dampened response to ligand in ER-positive disease, then the ratio of LXR to corepressor should predict target gene expression. As expected, we found that the ratio of LXR to all three corepressors was significantly higher in ER-negative tumours compared to ER-positive (Figure 4e; two-way ANOVA: *p* < 0.0001) supporting the proposal that LXR is better able to activate its transcriptional targets in ER-negative disease. Furthermore, whilst there was no correlation between LXRA and ABCA1 in the ER-positive cohort (Figure 2e), when assessing a correlation between ABCA1 expression and the ratio of LXRA/NCOR1 we found a significant positive correlation (Pearson correlation: R = 0.27, *p* < 0.0001; Figure 4f). Although the expression of APOE was significantly correlated with LXRA alone (Figure 2f), when the corepressors were included in the analysis the strength of correlation increased (NCOR1 r = 0.32; NCOR2 r = 0.36; LCOR r = 0.44 (Figure 4g)). Surprisingly, the strength of correlation between target gene and LXR was not improved by the addition of CoR expression in ER-negative disease ratio analysis. As a control we performed the same analyses for LXRB/NR1H2 and found no correlation with ABCA1 nor APOE in either subtype with any LXRB/CoR ratio (data not shown). These analyses reveal that the ratio of LXR to CoR is strongly correlated with target gene expression in all breast cancers analysed. These data are consistent with the hypothesis that the relative expression of LXRA to corepressors is the determinant of target gene responsiveness to ligand and that differences in this ratio between BCa subtypes determines their ability to dynamically respond to changes in cholesterol metabolic flux.

### 3.4. Removal of Corepressors Equalizes the Response of ER-Negative and ER-Positive Cell Lines to Ligand

Since corepressor expression and LXR transcriptional response to ligand appeared to be associated, we tested if knock-down of the corepressors in ER-positive cells equalized the response to ligand between MCF7 and MDA-MB-468 cells. Furthermore, basal expression of target genes should become elevated in knock-down cells owing to derepression following loss of corepressor activity. To this end we impaired NCOR1/NCOR2 or LCOR expression in luciferase reporter MDA-MB-468 and MCF7 cells using silencing RNA (50%–80% knock-down was observed for all corepressors in both cells lines (Figure S8)), followed by treatment with 26-OHC or vehicle control.

**Figure 5:**
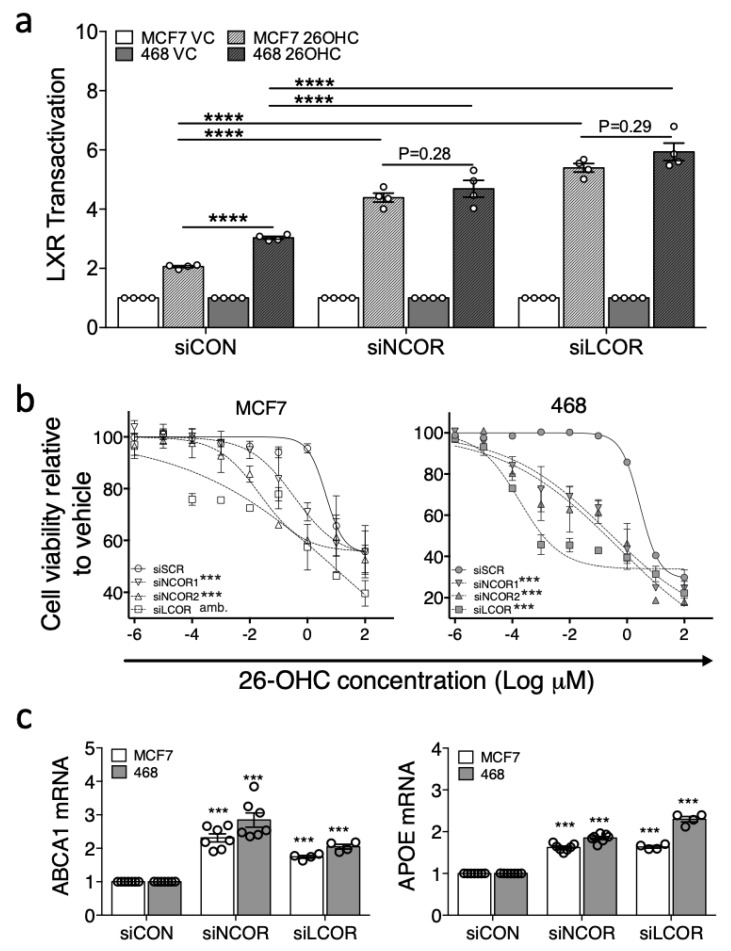
Corepressors determine differential response of cell lines to 26-OHC. NCOR or LCOR, were knocked-down in LXR-luciferase reporter MCF7 and MDA-MB-468 cells and treated with vehicle control (VC) or 26-OHC (10 μM) for 16 h (**a**). Endogenous LXR activity was determined after knockdown for ABCA1 (**b**) and APOE (**c**). Response to 26-OHC was assessed following corepressor knockdown by MTT (**d**). One-way ANOVA (a–c) and non-linear regression (d) were used to test for significant differences. *** *p* < 0.001, **** *p* < 0.0001, amb. = curve fit was ambiguous.

Under control conditions (siCON) LXR activation in response to 26-OHC was, as expected, significantly higher in MDA-MB-468 than MCF7 cells (two-tailed student’s *t*-test: *p* < 0.0001 (Figure 5a)). Knock-down of NCOR or LCOR however, significantly enhanced the transcriptional response to ligand in both cell lines (two-way ANOVA: *p* < 0.05 (Figure 5a)) and led to equivalent transcriptional responses to 26-OHC in MCF7 and MDA-MB-468 cells (paired two-tailed *t*-test: siNCOR *p* = 0.28; siLCOR *p* = 0.29 (Figure 5a)) suggesting corepressor expression was an important factor in determining the differential response of these two cell types to 26-OHC. This observation was recapitulated at the phenotype level, as corepressor knock-down led to a significantly enhanced attenuation of cell viability in response to 26-OHC treatment (non-linear regression comparison of fits: *p* < 0.0001 for both cell lines (Figure 5b)). When the basal expression of canonical LXR target genes were measured, we observed elevated expression of both ABCA1 and APOE with corepressor knock-down relative to control treated cells, again in both cell lines (two-way ANOVA: *p* < 0.0001 (Figure 5c)). In summary, these knock-down experiments support the hypothesis that corepressors are important determinants of the differential transcriptional activity of LXR between BCa subtypes.

## 4. Discussion

The importance of cholesterol and cholesterol metabolism in breast and other cancers is increasingly appreciated. The purpose of this study was to clarify whether the activity of LXR was different between ER-positive and ER-negative BCa and identify factors that may be responsible for any difference. We established that expression of LXR’s regulatory factors were skewed towards a transcriptionally poised state in ER-negative disease, but towards ligand insensitivity in ER-positive disease. Furthermore, LXRA expression positively correlated with that of its target genes in ER-negative tumours but not in ER-positive disease. Nuclear corepressor expression was elevated in primary ER-positive disease and experimental manipulation in vitro established they were critical in suppressing the response to ligand in ER-positive BCa. These data indicate that ER-negative tumours are particularly sensitive to elevated cholesterol and, given the increasing appreciation of the role of LXR signalling in BCa, potentially explain why ER-negative disease is more likely to be altered by cholesterol lowering interventions than ER-positive disease [39,40,41].

NR repression is a mechanism to overcome anti-proliferative actions in a range of cancer types including prostate [9,10] and bladder [11]. We observed anti-proliferative actions of the scOHC-LXR signalling axis, but it was surprising that a permissive anti-proliferative LXR signalling environment was retained in the more aggressive ER-negative BCa subtype. In our study we evaluated T47D, MCF7, MDA-MB-468 and MDA-MB-231 cells, all of which responded in vitro consistently with in vivo observations from primary breast tumours; the ER-positive models had a dampened transcriptional response to LXR ligands compared to ER-negative. A previous report indicated that ER-positive MCF7 and T47D cells were more sensitive to LXR induced G0/G1 arrest than ER-negative MDA-MB-231 cells [32], but at the same time indicated, like us, that LXR stimulation led to higher induction of *ABCA1* in ER-negative cells than in ER-positive ones. This discrepancy in sensitivity between cell cycle and direct transcriptional regulation, probably reflects the fact that the synthetic ligands used in the cell cycle arrest analysis are not oestrogenic, whereas in our study we observe the opposing actions of scOHCs on ER and LXR in ER-positive cells. As we demonstrate here, there are differences in NR biology between BCa subtypes beyond ER/PR expression, and responsiveness to LXR ligands is influenced by corepressor expression and indicates differential cholesterol metabolism between BCa subtypes.

Retention of LXR signalling in some tumour types suggests a selective advantage that compensates for the anti-proliferative actions of the scOHC-LXR axis [32]. The oxysterol signalling axis is emerging as a route through which ER-negative BCa metastasis may occur [17]. It would be interesting to determine if the early tumour development requires repressed LXR activity so as to impair its anti-proliferative actions, and contrast with a return to LXR activation in later stage disease to support migration. Consistent with this is the observation that 25-OHC is elevated in the serum of breast cancer patients at relapse compared to those with primary disease [18].

The differences we observed in LXR activity between subtypes expand on previous observations that NR cofactors could usefully serve as therapeutic biomarkers, which are targetable through epigenetic drugs (e.g., HDAC inhibitors that impair their epigenetic transcription silencing targets and that are recruited by NCOR1 and NCOR2) aimed at reinstating pre-cancer gene expression patterns and responsiveness. NCOR1 expression, for example, was found to be an independent and favourable prognostic marker in a mixed BCa cohort [42]. This may in part be explained by Tamoxifen’s dependence on NCOR1 recruitment to and repression of ER target genes in ER-positive BCa [43]. In therapy naïve ER-positive tumours our data suggest corepressor levels are high, perhaps to prevent LXR (and indeed other NRs such as VDR) from driving anti-proliferative transcriptional programs. The impact of high corepressor expression on scOHC dependent ER activity may also be important. Several scOHCs are oestrogenic and pro-proliferative when liganded with ER, indicating that elevated corepressor expression may serve to impede scOHC-ER dependent proliferation.

LCOR is also of therapeutic and prognostic interest as its recruitment to promoters by agonists can repress gene expression rather than activate [44]. It is tempting to link two of our observations; *LCOR* levels were significantly higher in ER-positive disease (and in MCF7 cells), and agonist treatment led to repression of *APOE* (Figure 2d) in MCF7 cells only. LCOR expression has previously been reported to be associated with better survival in BCa patients [37], particularly if nuclear localization is considered [45], which presumably reflects its inhibitory actions on oestrogen receptor signalling. Further research is required to understand if manipulation of LCOR expression can mediate the selective modulation of LXR ligand function, as our observations of *APOE* transcription could suggest.

The methodology we employed to identify an unbiased panel of LXR target genes, which was then used to test if LXR was transcriptionally active or repressed in different tumour types, has potentially identified a large set of novel LXR target genes. Our analysis combined ChIP-Seq data from multiple cell types with validation of potential targets by assessing expression in primary BCa samples and induction analysis in vitro. This approach resulted in multiple apparently novel LXR target genes being identified, with three out of three validated by qPCR. The first of these LCP2, has been reported as differentially expressed between primary and metastatic colorectal cancer [46] and is a prognostic biomarker for colorectal cancer patients [47]. *TNFRSF1B* expression has been linked to increased BCa risk [48,49] and to chemotherapy resistance via enhanced AKT signalling and PARP mediated DNA repair [50]. DOK2 has tumour suppression roles in several cancer types as it impairs MAPK activation and loss of its expression is associated with poor survival in lung adenocarcinoma [51], whilst in BCa greater DOK2 expression is associated with significantly longer disease free survival [52]. These possible LXR targets, as well as others in ST1 require further evaluation to ascertain the extent to which they, through aberrant cholesterol metabolism and LXR signalling, may influence tumour biology.

It is interesting to note that many of lifestyle factors reported by the World Cancer Research Fund’s Continuous Update Project [53] that associate with BCa, are body composition metrics and nutritional parameters that are directly associated with LDL-C, a key precursor of scOHCs. LDL-C, Obesity, Waist-Hip-Ratio and Waist Circumference are associated with incidence and survival of BCa [54,55] and clinically recommended diets/lifestyle changes that lower LDL-C (e.g., high fish-oil and carotenoid intake, the Mediterranean Diet, reduced animal calorie intake), protect against BCa and relapse, particularly in the hormone receptor negative setting [41,53,56,57]. Pharmacological manipulation of LDL-C with lipophilic statins improves BCa survivorship [39], specifically reducing early (<4 years) relapse events [40], again, a feature typical of ER-negative disease. Our data are consistent with the hypothesis that ER-negative tumours are more sensitive to changes in systemic cholesterol flux; future work should clarify if dietary or pharmacological suppression of scOHC signalling could modify disease prognosis.

## 5. Conclusions

In this current study, scOHC were confirmed as natural LXR agonists in BCa cell lines, and we observed that their activity is regulated to a large extent by corepressors. This is the first demonstration that transcriptional activation of LXR target genes by scOHCs may be dependent on tumour-subtype specific expression of corepressors. A combination of mechanistic and clinical trial studies should help confirm the relevance of the data described here in people, and would allow further exploration of LXR as a potential therapeutic target that links dietary and lifestyle regulation of cholesterol metabolism with cancer progression and survival.

## Supplementary Materials

The following are available online at https://www.mdpi.com/2072-6643/11/11/2618/s1, Table S1: Cistrome derived LXRA promoter occupancy binding scores for genes selected for correlation analysis, Figure S1: Flow diagram of generation of LXR target gene list, Figure S2: ER-negative (MDA-MB-468 and MDA-MB-231) and ER-positive (MCF7 and T47D) cell lines were stably transfected with LXRA-Luciferase reporter constructs and treated with the synthetic LXR agonist GW3965 1 μM 16 h, Figure S3: (a) HEPG2 liver control cells respond robustly to ligand in a similar manner to the ER-negative cell reporter constructs. The HEPG2 cell line was stably transfected with LXRALuciferase reporter constructs and treated with indicated LXR ligands at indicated concentrations. (b) siLXRA prevents the LXR-luciferase assay from responding to 26-OHC or 24,25-EC, Figure S4: ABCA1 expression is LXR dependent. (a) ER-negative (MDA-MB-468) and ER-positive (MCF7) cell lines were treated with a panel of ligands (Vehicle control, GSK2033 (1 μM), 26-OHC (10 μM) and a combination of 26-OHC (10 μM) + GSK2033 (1 μM) for 16 h. (b) Cells treated with siCON or siLXRA were assessed for gene expression and ABCA1 levels were reduced in both MCF7 and MDA-MB-468 in the absence of LXRA. Gene expression of the canonical LXR target genes was assessed by qPCR using ΔΔcT method against HPRT1. Data are means of 3–4 independent replicates with SEM, Figure S5: LXR canonical target genes do not correlate with LXRB/NR1H2 in ERnegative tumours. mRNA-Seq data were obtained for 81 ER-negative and 234 Luminal A tumours (TGCA) and expression of the LXR target genes ABCA1 and APOE were correlated with NR1H2/LXRB expression and assessed for linear regression, Figure S6: Recruitment of LXRA to canonical (a) and novel (b) target gene promoters after GW3965 treatment. The promoter regions of the LXR canonical target genes ABCA1 and APOE (a) and the novel target genes TNFRSF1B, LCP2 and DOK2 (b) were assessed for LXRA recruitment in macrophages 24 h post exposure to vehicle control and the LXR ligand GW3965. Peak intensity between treatments are displayed highlighting the changes in binding of LXRA within a 10 kb promoter region, Figure S7: Publicly available datasets of Luminal A (25 cell lines, including MCF7) and ER-negative (25 cell lines, including MDA-MB-468) expression of the corepressors NCOR1, NCOR2/SMRT and LCOR were analysed for differential expression between BCa subtypes. CoR expression is presented as box and whisker plots with median, inter-quartile and 10-90 percentiles shown. MCF7 and MDA-MB-468 cell line expression are highlighted. Statistical significance was established using two-tailed Mann Whitney-U tests. *p<0.05, Figure S8 Validation of NCOR1, NCOR2 and LCOR knock down. NCOR1 and NCOR2 in combination and LCOR alone were silenced in ER-positive LXR-luciferase cell cultures (MCF7) and ER-negative LXR-luciferase cell cultures (MDA-MB-468). Gene expression of the corepressor NCOR1 (a), NCOR2 (b) and LCOR (c) were assessed by qPCR (b) 36 h post silencing using ΔΔcT (normalised to HPRT1). Statistical analysis was established using two-way ANOVA and is representative of 2–3 independent replicates.
