# Supplementary material for: ER-Negative Breast Cancer Is Highly Responsive to Cholesterol Metabolite Signalling
Source: Nutrients. 2019 Nov 1;11(11):2618. doi: 10.3390/nu11112618 (PMC6893441; doi:10.3390/nu11112618)
Supplement: Supplementary file 1 [file nutrients-11-02618-s001.pdf]

ST1

	Oishi, Y., *et al*, 2017.				Galhardo, M., *et al.*, 2013.	Savic, D., *et al.*, 2016.	
	Macrophage No Treatment	Macrophage GW3965	Macrophage KLA6H	Macrophage KLA1H	Adipocyte	Colorectal Cancer GW3965 48H	Colorectal Cancer GW3965 2H
**A130077B15RIK**	1.88	**3.512**	2.019	**3.097**	0	0	0
**ABCA1**	**3.58**	3.29	**3.865**	**3.332**	**0.948**	2.382	1.981
**ABCG1**	2.58	2.922	2.266	**3.073**	**1.652**	2.0945	2.21
**ABCG5#**	0	0.153	0	0	0	0.858	0.996
**ABCG8#**	0	0.155	0	0	0	0.853	0.995
**ABLIM3**	0	0	0	0	0	**3.5665**	**3.1305**
**ACACA#**	0.345	1.154	0.027	0.027	0.604	1.387	1.094
**ADAMTSL4**	**3.818**	**4.073**	**3.281**	1.974	0	1.1115	1.3845
**AFF1**	**3.423**	**3.556**	1.849	**3.958**	0	2.3825	1.761
**AIM1L**	0.396	0.437	0.343	0.343	0	**3.3815**	**3.023**
**AMZ2**	**3.215**	2.785	**3.644**	**3.95**	0	0.847	0.4725
**ANKRD22**	0.683	1.636	1.24	0.706	**0.603**	**3.383**	2.754
**AP2S1**	0.725	0.728	0.626	0.726	0	**3.0865**	**3.1435**
**APBB1IP**	**3.033**	**3.747**	2.462	2.407	0	0.152	0.107
**APOE#**	1.339	3.268	0.7	1.648	0.727	1.531	1.6
**ARHGAP25**	**3.596**	**4.148**	2.676	**3.394**	0	0.643	0.319
**ARHGAP26-AS1**	0	0	0	0	0	**2.667**	**2.9995**
**ASPH**	1.855	**4.37**	**2.822**	2.944	0	2.15	2.1115
**B330016D10RIK**	2.336	3.478	**3.71**	**3.18**	0	0	0
**BCL3**	2.421	3.108	**3.292**	2.807	0	**3.878**	**4.512**
**BMF**	1.58	2.439	2.388	1.242	0	**3.9315**	**3.408**
**C14ORF182**	0	0	0	0	0	**2.119**	**2.028**
**C1QTNF1-AS1**	0	0	0	0	0	**3.4415**	**3.3795**
**C6ORF222**	0	0	0	0	0	**3.133**	**3.5385**
**CAPN2**	1.459	1.931	1.558	1.661	0	**3.3725**	**3.889**
**CAPN5**	0.508	1.931	1.568	1.091	0	**3.1095**	**4.036**
**CCL3**	**2.979**	2.949	**3.086**	**3.237**	0	0.352	0.2015
**CCL6**	**3.403**	**4.196**	**3.416**	**3.304**	0	0	0
**CCL9**	**3.16**	**3.875**	**2.839**	**3.578**	0	0	1.593
**CD14**	**3.055**	3.463	2.708	**3.845**	0.486	2.247	2.3265
**CD300A**	**4.041**	**4.719**	1.777	**3.602**	0	1.6455	1.367
**CD300C**	**3.206**	2.986	1.799	**3.069**	0	0.1225	0.074
**CD300LB**	**3.664**	3.376	1.664	**3.325**	0	0.217	0.169
**CD5L#**	2.037	2.554	1.251	1.567	0	0.135	0.079
**CETP#**	0	0	0	0	0	1.172	1.269
**CLCF1**	1.453	3.264	**3.114**	2.473	0	2.672	**3.299**
**CTDSP1**	**3.398**	2.552	**2.789**	2.027	0	2.346	2.4175
**CYP7A1#**	0.149	0.171	0.115	0.231	0	0.615	0.647
**CYTH4**	**3.783**	3.311	2.335	**3.074**	0	0.6645	0.497
**DDX47**	1.023	0.88	0	0	0	**3.582**	**3.348**
**DNAJC17**	0.959	0.19	0.152	0.756	0	**3.0395**	**2.9305**
**DOK2**	1.759	2.843	**2.747**	**3.306**	0	0.512	0.512

DUSP1	2.593	2.63	2.496	2.319	0	3.9665	3.3765
E230016K23RIK	3.231	3.942	2.995	3.607	0	0	0
E230025N22RIK	3.103	3.22	2.269	3.469	0	0	0
EEPD1#	0.989	1.309	1.345	1.04	0	2.009	1.569
EHD1	1.794	2.368	1.529	2.866	0	3.472	2.802
EHF	0	0	0	0	0	2.903	3.8045
ELF3	0.744	1.155	0.744	1.314	0	3.346	3.42
ELOVL5#	2.525	1.897	1.742	2.059	0.4	0.428	0.455
FAM83E	1.473	1.743	0.942	1.7	0	3.3635	3.515
FASN#	0.705	0.9	0.627	0.677	0.588	1.416	1.836
FCGR2B	2.136	4.169	3.928	2.687	0	0.4555	0.409
FCRLA	2.328	3.889	2.757	2.435	0	1.3235	0.899
FEM1A	2.693	3.623	3.101	2.425	0.595	1.7125	1.3795
FIZ1	0.256	1.773	0.79	1.105	0	3.292	3.169
FLOT1	1.678	2.642	2.549	2.901	0	3.189	3.308
FYB	3.254	3.948	3.119	3.076	0	0.5905	0.7515
GM13031	2.687	3.679	2.792	1.809	0	0	0
GM14005	1.858	2.989	3.501	4.307	0	0	0
GM19510	3.025	3.425	4.473	3.341	0	0	0
GM2848	1.679	3.618	2.845	2.845	0	0	0
GPRC5C	1.231	2.793	0.428	2.113	0	3.229	3.4955
HCG27	0	0	0	0	0	3.5385	3.1945
IER3	1.699	2.677	2.639	2.976	0	3.23	3.343
IL18BP#	0.452	2.659	0.81	0.472	0	0.423	0.603
IL1B	1.506	2.671	2.748	3.021	0	2.1675	2.4235
IL21R	3.381	4.466	3.09	3.984	0	0.4955	0.3435
IL9#	0	0.5	0	0	0	0.645	0.494
IRF8#	2.047	2.534	2.025	2.718	0	1.244	0.636
ITGB6	0.059	0.029	0.033	0.209	0	3.1685	3.287
JUP	1.07	1.823	0.566	0.498	0	2.966	3.3015
KRT7	0.383	0.667	0.759	0.653	0	3.4625	3.416
LCP2	2.711	3.765	2.77	3.961	0	0.5135	0.0135
LINC00880					0	3.545	3.819
LOC100503496	2.582	2.471	2.755	3.229	0	0	0
LOC100506499	0	0	0	0	0	1.94	3.8165
LOC101928093	0	0	0	0	0	3.1675	3.0705
LOC254099	0	0	0	0	0	1.9805	3.4
LOC731656	0	0	0	0	0	3.606	3.6505
LPCAT3#	1.199	1.924	1.475	1.285	0.711	1.526	1.836
LPL#	1.28	1.298	1.75	1.746	0.385	0.017	0.017
LTBR	1.653	2.152	1.422	1.884	0	3.2635	3.4655
LY9	3.46	3.665	1.806	2.926	0	0.7805	0.756
MAPK6	0.812	1.723	1.028	1.419	0.135	3.7225	3.331
MCL1	4.279	4.495	3.655	2.297	0	1.5605	1.2855
MGRN1	3.456	4.423	3.16	3.353	0	1.448	1.283
MIR101C	4.51	2.906	4.317	3.499	0	0	0
MIR192	1.641	2.166	1.541	2.528	0	3.733	2.9125
MIR194-2	1.635	2.16	1.535	2.518	0	3.7315	2.912
MIR26B	3.394	2.451	2.787	1.977	0	2.354	2.442
MIR6076	0	0	0	0	0	2.779	3.6895
MIR6750					0	3.508	2.5795
MIR8085	0	0	0	0	0	3.497	3.9775
MPEG1	3.952	5.67	2.183	3.195	0	1.7955	1.6755
MSL1	1.905	3.241	3.265	3.088	0	2.2855	1.981
MYLIP#	0.663	0.958	0.93	1.614	0.58	1.531	1.406

**N4BP1**	3.185	3.073	2.214	3.151	0	1.591	1.363
**NDST1**	**4.492**	**3.72**	1.577	2.665	0	1.9775	1.552
**NINJ1**	**2.954**	**3.484**	2.441	2.619	0	2.606	1.442
**NPC1#**	0.571	1.038	0.559	0.471	0	1.152	0.728
**NPC2#**	1.559	2.42	1.369	2.202	0	0.143	0.036
**NR1D1**	1.991	3.211	**3.659**	**3.259**	0	2.442	2.3405
**OMP**	0.385	0.898	1.23	0.515	0	**3.276**	**3.808**
**PDE4B**	2.176	2.776	**3.329**	**3.392**	0	0.017	0.03
**PGC**	1.275	1.949	0.416	1.486	0	**3.287**	**3.3635**
**PIGC**	1.298	1.549	0.434	1.62	0	**3.406**	**3.6525**
**PIK3CG**	2.286	3.206	**3.416**	**3.956**	**0.552**	0.742	0.519
**PILRA**	**3.799**	2.98	**3.319**	2.347	0	0.5855	0.296
**PILRB1**	**3.737**	2.891	**3.06**	2.359	0	0.4225	0.3405
**PLA2G7**	2.649	3.452	**2.784**	**3.047**	0	0.4055	0.3365
**PLAU**	**2.942**	2.493	2.301	**3.535**	0	2.1345	1.938
**PLTP#**	2.915	2.374	1.912	1.709	0	1.422	1.985
**PLXND1**	2.017	**3.61**	2.541	**3.5**	0	0.863	0.9205
**PNPLA2**	**4.061**	**3.586**	1.942	2.232	0	1.9595	2.0065
**PPM1N**	1.587	2.016	1.709	**3.105**	0	**2.8845**	2.3235
**PRKCD**	**2.956**	**3.829**	**3.205**	2.869	0	2.33	2.154
**PTK2B**	**3.399**	3.037	2.382	**3.366**	0	1.8775	1.7165
**RALB**	1.796	1.457	0.185	1.797	0	**3.4605**	**3.0205**
**RALGDS**	2.782	3.06	2.138	**3.286**	0	**3.2175**	2.6305
**RHOV**	1.876	1.752	1.271	1.717	0	**3.259**	**3.282**
**RNF145#**	1.613	1.256	1.64	1.758	0.606	0.968	1.818
**RPL18**	1.654	1.963	1.069	1.79	0	**3.2965**	**3.362**
**RPLP2**	**3.605**	**3.533**	1.682	2.159	0	1.7275	1.846
**RTN2**	1.52	2.081	1.666	**3.041**	0	**2.9505**	2.3765
**SCD1#**	1.335	1.886	0.937	1.408	1.298	1.719	1.521
**SCD5#**	0	0	0	0	0	0.015	0.015
**SCNN1A**	1.589	2.267	1.278	1.862	0	**3.2685**	**3.4695**
**SDHB**	2.85	**3.865**	**3.069**	1.901	0	1.379	0.693
**SLA**	**3.115**	2.811	1.966	**3.294**	0	0.6185	0.229
**SLC2A4**	2.275	1.766	1.675	1.278	**0.596**	**3.204**	1.9825
**SLFN2**	2.381	**3.811**	2.057	**3.409**	0	0	0
**SMAD3**	1.235	1.206	1.921	1.875	0	**3.4055**	**3.695**
**SMIM5**	0	0	0	0	0	**3.5235**	**3.638**
**SMIM6**	0	0.027	0.296	0.256	0	**3.5365**	**3.689**
**SNAR-E**	0	0	0	0	0	**3.46**	**3.5265**
**SNORA52**	**3.652**	**3.519**	1.711	2.158	0	1.761	1.8605
**SNRNP35**	**2.964**	3.157	2.319	**3.293**	0.014	1.2415	1.4695
**SOCS3**	1.542	2.541	1.914	**3.162**	**0.662**	2.6945	2.499
**SPACA4**	1.24	1.469	0.784	1.607	0	**3.3715**	**3.5865**
**SPHK2**	1.658	2.131	1.146	1.884	0	**3.3025**	**3.367**
**ST6GAL1**	**3.009**	**3.593**	**3.023**	2.83	0	0.2845	0.684
**SULT2B1**	0.644	0.603	0.199	1.145	0	**3.8135**	**3.968**
**SYK**	1.535	**3.545**	**2.991**	**3.575**	0	0.7315	0.945
**SYNJ2**	**3.049**	**3.515**	1.844	2.089	0	1.8535	2.55

**TANK**	1.808	3.528	**1.457**	**1.104**	0.594	**1.9025**	**2.368**
**TEC**	**3.44**	**3.953**	2.71	**3.579**	0	1.686	1.1565
**TFEB**	**2.443**	2.668	1.567	1.989	0	**3.4055**	**3.4555**
**TGFB1**	**3.797**	3.099	**2.846**	2.793	0	0.707	1.0155
**TGFBI**	**2.365**	**3.847**	1.462	1.411	**0.543**	2.4415	2.4185
**TGM2**	**3.658**	**3.647**	**4.156**	2.886	0.172	**2.8655**	3.179
**THEMIS2**	**2.897**	**3.848**	1.854	**3.383**	0.067	1.078	1.092
**TICAM1**	**2.225**	**3.758**	**2.829**	2.435	0.12	1.599	1.0705
**TLR4[#]**	2.621	1.719	2.704	1.723	0	0.778	0.676
**TMCO6**	**2.486**	3.059	1.999	**3.309**	**0.602**	2.3185	2.3785
**TMEM154**	**4.251**	**3.55**	2.661	2.319	0	0.461	0.3375
**TMEM185B**	**1.121**	1.471	0.434	1.121	0	**3.539**	**2.7235**
**TMEM72**	**1.468**	2.243	**3.042**	**4.138**	0	0.7675	1.1515
**TNFAIP2**	**2.33**	2.221	2.022	1.991	0	**3.645**	**3.947**
**TREML2**	**2.112**	**4.558**	1.575	**3.257**	0	0.586	1.078
**TRIM31**	**0.661**	0.79	0.646	0.648	0	**3.652**	**4.728**
**TRIM40**	**0.514**	0.691	0.503	1.091	0	**3.4775**	**4.378**
**TRNFRSF1B**	**3.183**	**3.911**	**4.32**	**3.843**	0	1.416	0.891
**TRP53COR1**	**1.885**	2.604	**2.883**	**3.262**	0	0	0
**TXNDC2**	**2.661**	**4.012**	**2.997**	**3.222**	0	2.202	2.0835
**UBALD1**	**3.179**	**4.162**	**3.048**	**3.167**	0	1.29	1.3075
**VASP**	**1.955**	2.23	1.97	**3.204**	0	**3.0555**	2.4965
**ZFYVE19**	**0.956**	0.189	0.152	0.754	0	**3.034**	**2.93**
**ZNF524**	**0.261**	1.821	0.811	1.132	0	**3.296**	**3.1485**
**ZNF598**	**1.289**	0.905	1.344	1.662	0	**3.1385**	**2.867**
**ZNRF1**	**1.883**	1.862	**4.023**	**3.055**	0	1.01	1.0075
**5031414D18RIK**	3.344	**4.637**	1.512	2.904	0	0	0

SF1

The top 100 genes with the highest LXR binding scores from each of the seven ChIP-Seq datasets and their binding scores were collated.

↓

148 genes that appeared on two or more lists were included for further analysis.

↓

11 genes were removed as they were from mouse datasets and had no human homologue.

↓

26 genes were removed as they were not expressed in the TCGA breast cancer datasets.

↓

An additional 24 genes that are established LXR targets but did not necessarily reach cut-off scores were added.

↓

The final list of 135 genes was assessed for correlation with NR1H3/LXRA mRNA using Pearson's correlation.

↓

A False Discovery Rate (FDR) of 1 % was applied.

↓

A Fisher's exact test used to establish if the number of genes correlating with NR1H3 was significantly different between the two subtypes.

SF2

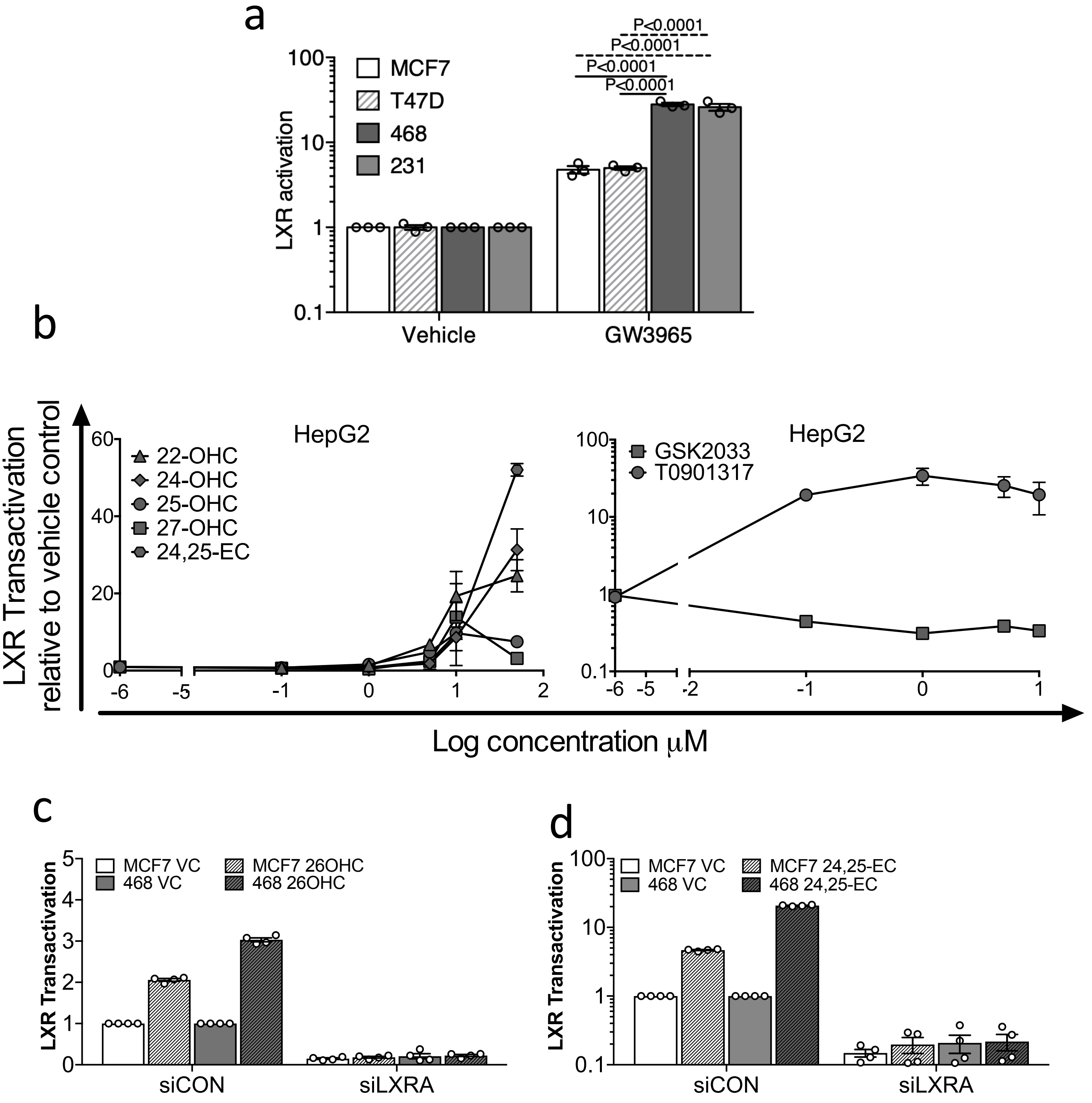
a
P<0.0001
P<0.0001
P<0.0001
P<0.0001
MCF7
T47D
468
231
LXR activation
Vehicle
GW3965
b
LXR Transactivation relative to vehicle control
HepG2
22-OHC
24-OHC
25-OHC
27-OHC
24,25-EC
HepG2
GSK2033
T0901317
Log concentration μM
c
LXR Transactivation
MCF7 VC
MCF7 26OHC
468 VC
468 26OHC
siCON
siLXRA
d
LXR Transactivation
MCF7 VC
MCF7 24,25-EC
468 VC
468 24,25-EC
siCON
siLXRA

SF3

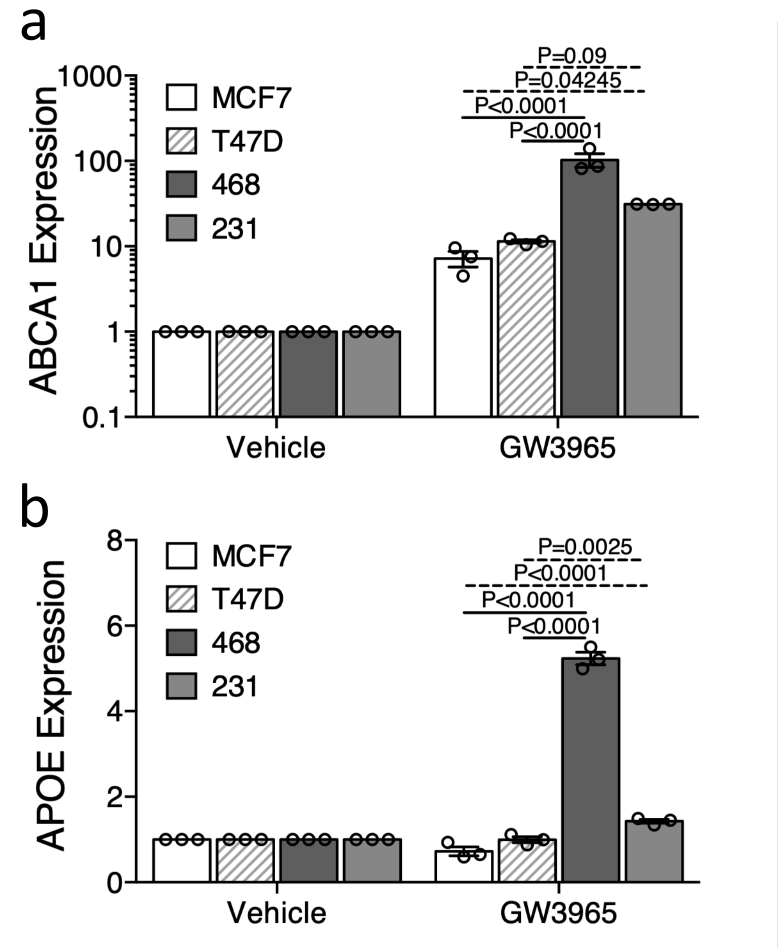
a
P=0.09
P=0.04245
P<0.0001
P≤0.0001
1000
100
10
1
0.1
ABCA1 Expression
MCF7
T47D
468
231
Vehicle
GW3965
b
P=0.0025
P<0.0001
P<0.0001
P≤0.0001
8
6
4
2
0
APOE Expression
MCF7
T47D
468
231
Vehicle
GW3965

SF4

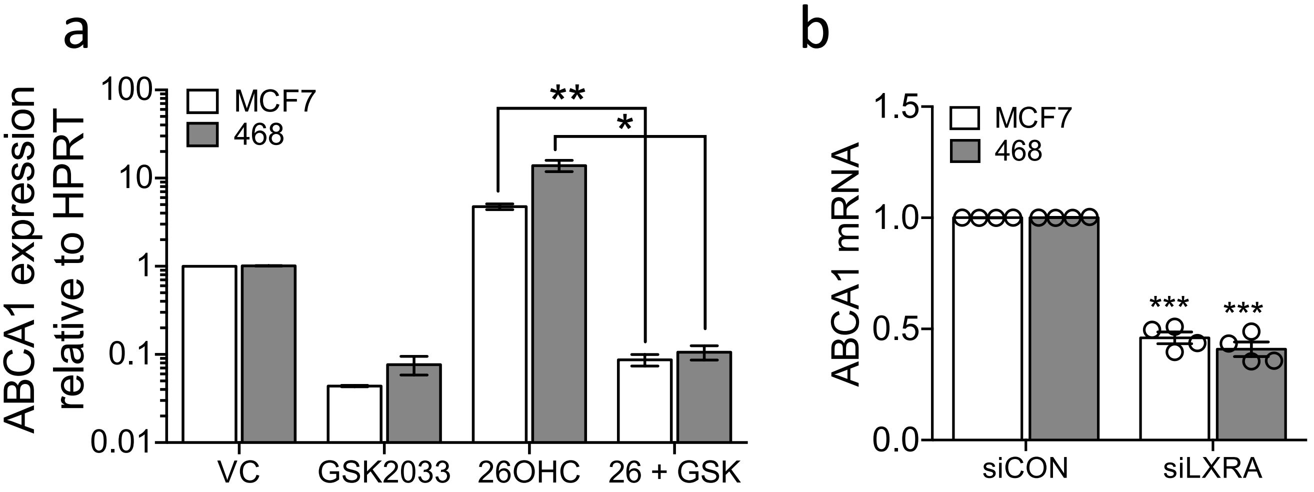
a
ABCA1 expression relative to HPRT
100
10
1
0.1
0.01
MCF7
468
**
*
VC
GSK2033
26OHC
26 + GSK
b
ABCA1 mRNA
1.5
1.0
0.5
0.0
MCF7
468
***
***
siCON
siLXRA

SF5

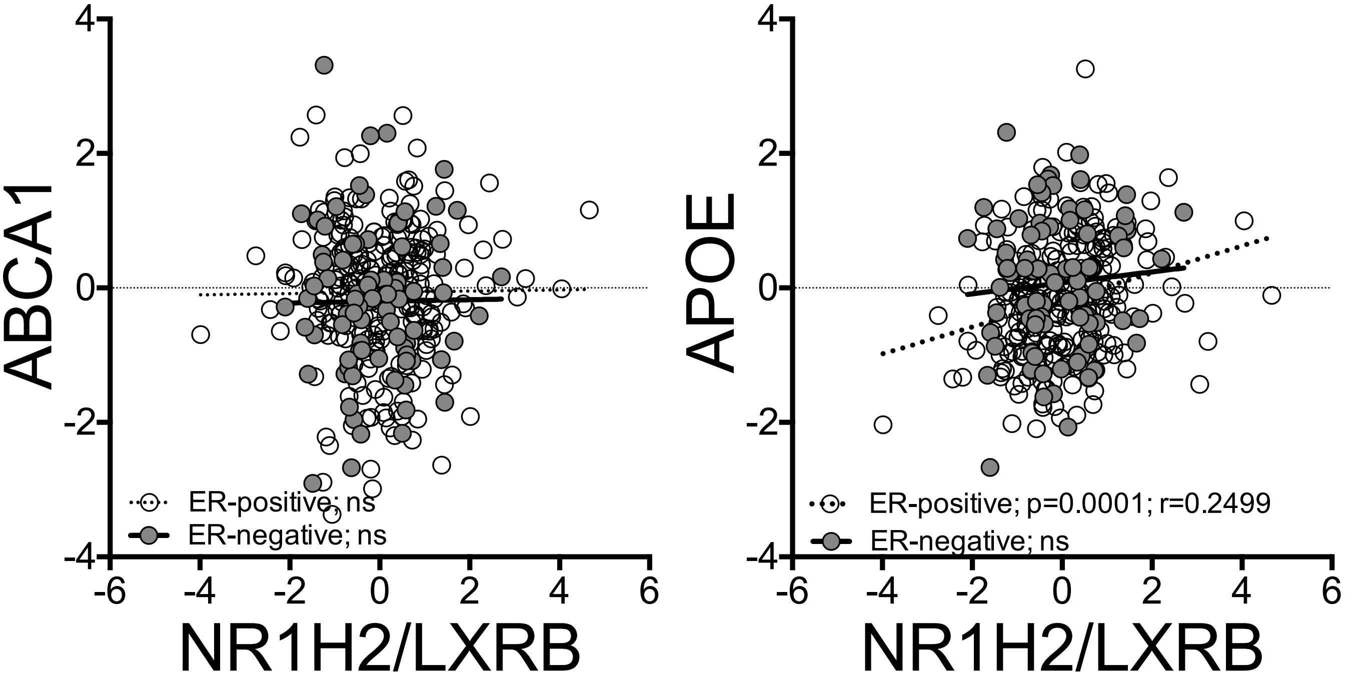

ABCA1
NR1H2/LXRB
ER-positive; ns
ER-negative; ns
4
2
0
-2
-4
-6
-4
-2
0
2
4
6
APOE
NR1H2/LXRB
ER-positive; p=0.0001; r=0.2499
ER-negative; ns

SF6

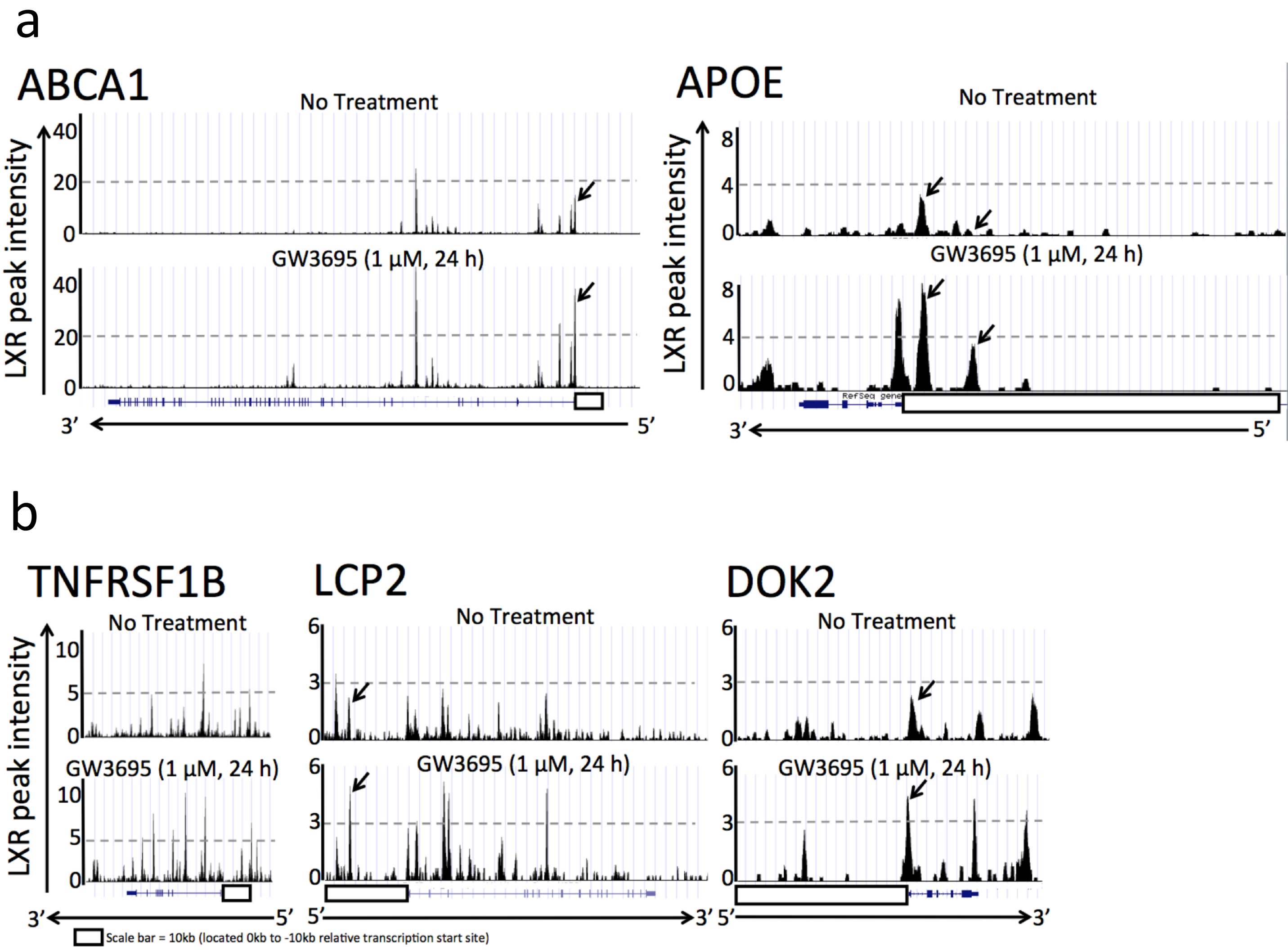
a
ABCA1
No Treatment
GW3695 (1 μM, 24 h)
LXR peak intensity
40
20
0
3'
5'
APOE
No Treatment
GW3695 (1 μM, 24 h)
LXR peak intensity
8
4
0
3'
5'
b
TNFRSF1B
No Treatment
GW3695 (1 μM, 24 h)
LXR peak intensity
10
5
0
3'
5'
LCP2
No Treatment
GW3695 (1 μM, 24 h)
6
3
0
5'
3'
DOK2
No Treatment
GW3695 (1 μM, 24 h)
6
3
0
5'
3'
Scale bar = 10kb (located 0kb to -10kb relative transcription start site)

SF7

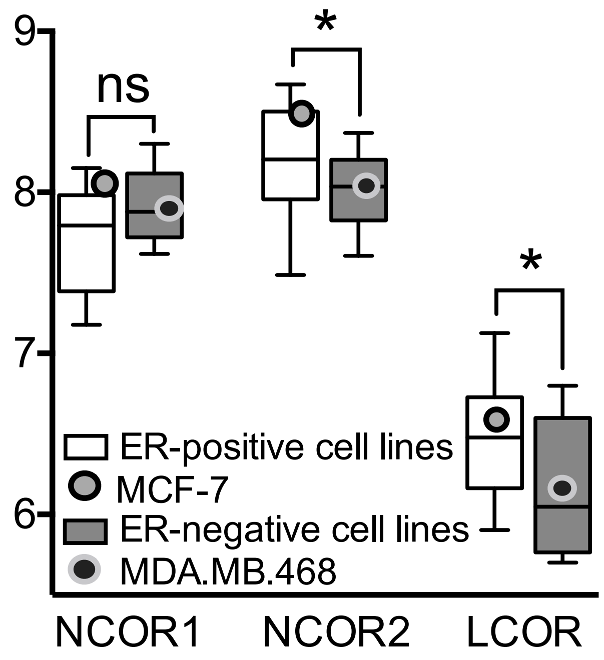
9
8
7
6
ns
*
*
ER-positive cell lines
MCF-7
ER-negative cell lines
MDA.MB.468
NCOR1
NCOR2
LCOR

SF8

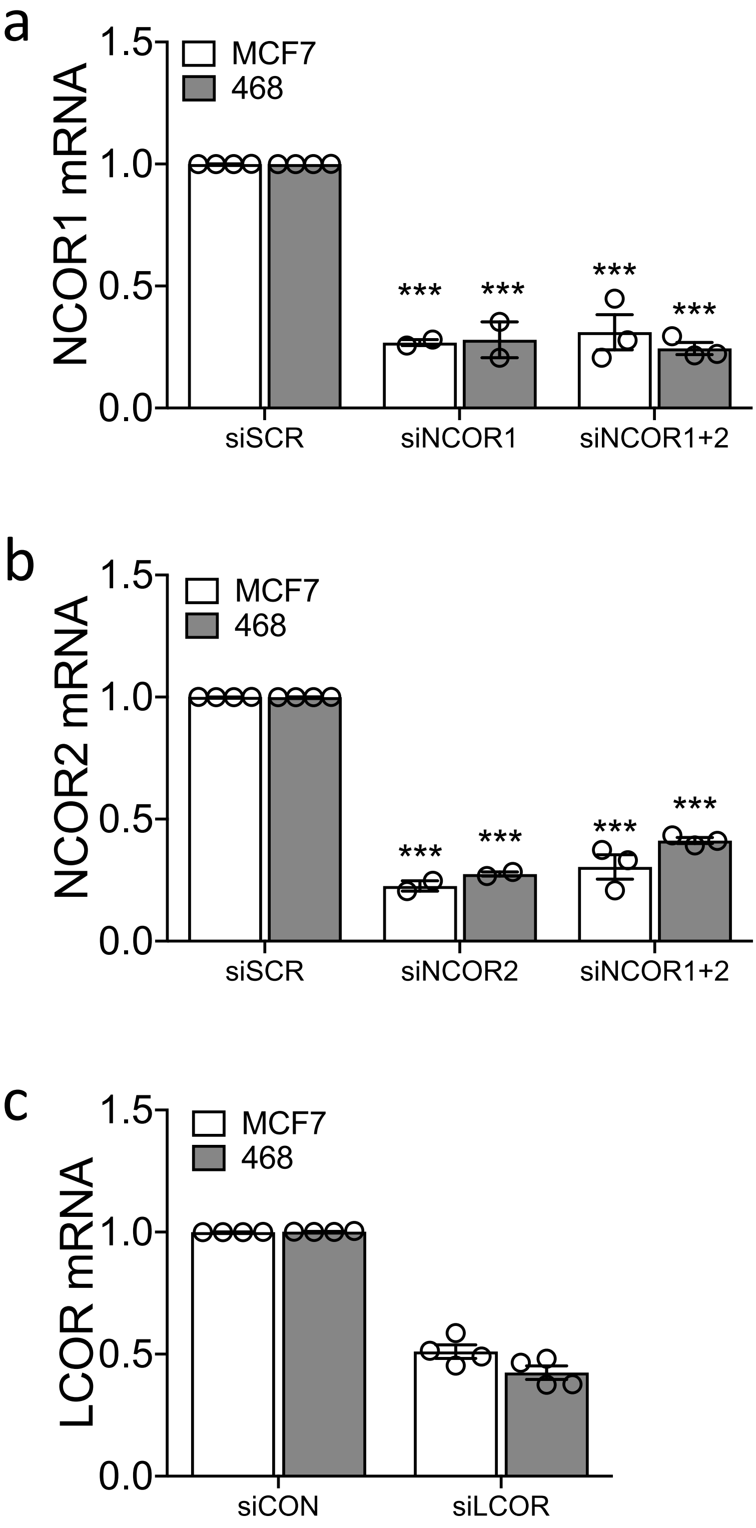
a
1.5
1.0
0.5
0.0
NCOR1 mRNA
MCF7
468
***
***
***
***
siSCR
siNCOR1
siNCOR1+2
b
1.5
1.0
0.5
0.0
NCOR2 mRNA
MCF7
468
***
***
***
***
siSCR
siNCOR2
siNCOR1+2
c
1.5
1.0
0.5
0.0
LCOR mRNA
MCF7
468
siCON
siLCOR

Supplementary Table 1 Cistrome derived LXRA promoter occupancy binding scores for genes selected for correlation analysis. Top 100 LXRA bound genes were assessed from seven LXRA Chip-Seq datasets deposited in cistrome before 01-03-2019. A binding score in bold indicates the gene was ranked in the top 100 for that dataset. Scores in grey were ranked below the 100 gene cut-off for that dataset, but appeared in the top 100 of at least two other datasets. Common top scoring genes (appeared in more than one dataset) were selected for further analysis. LXRA binding analysed by Oishi *et al.*, [29] was performed in primary mouse macrophages with no treatment, or synthetic LXR agonist, GW3965, for 24 h. Savic *et al.*, assessed LXRA binding in HT29 human colorectal cancer cells by exposure to GW3965 for 2 and 48 h [60]. Galhardo *et al.*, performed genome-wide ChIP-Seq profiles for LXRA in mature adipocytes [61]. Adipocytes sequenced were differentiated from a pre-adipocyte cell line sourced from a human Simpson-Golabi-Behemel syndrome (SGBS) patient. 24 common LXR target genes were included for analysis, even if they didn't reach the minimum cut-offs and are identified by #.
Supplementary Fig. 1 Flow diagram of generation of LXR target gene list.
Supplementary Fig. 2 ER-negative (MDA-MB-468 and MDA-MB-231) and ER-positive (MCF7 and T47D) cell lines were stably transfected with LXRA-Luciferase reporter constructs and treated with the synthetic LXR agonist GW3965 1 μM 16 h. Statistical analysis was assessed using two-way ANOVA (*p<0.05, **p<0.01, ***p<0.001) and data are derived from 3 independent replicates with SEM.
Supplementary Fig. 3 (a) HEPG2 liver control cells respond robustly to ligand in a similar manner to the ER-negative cell reporter constructs. The HEPG2 cell line was stably transfected with LXRA-Luciferase reporter constructs and treated with indicated LXR ligands at indicated concentrations. (b) siLXRA prevents the LXR-luciferase assay from responding to 26-OHC or 24,25-EC.
Supplementary Fig. 4 *ABCA1* expression is LXR dependent. (a) ER-negative (MDA-MB-468) and ER-positive (MCF7) cell lines were treated with a panel of ligands (Vehicle control, GSK2033 [1 μM], 26-OHC [10 μM] and a combination of 26-OHC [10 μM] + GSK2033 [1 μM]) for 16 h. (b) Cells treated with siCON or siLXRA were assessed for gene expression and ABCA1 levels were reduced in both MCF7 and MDA-MB-468 in the absence of LXRA. Gene expression of the canonical LXR target genes was assessed by qPCR using ΔΔCT method against HPRT1. Data are means of 3-4 independent replicates with SEM.
Supplementary Fig. 5 LXR canonical target genes do not correlate with *LXRB/NR1H2* in ER-negative tumours. mRNA-Seq data were obtained for 81 ER-negative and 234 Luminal A tumours (TGCA) and expression of the LXR target genes *ABCA1* and *APOE* were correlated with *NR1H2/LXRB* expression and assessed for linear regression.
Supplementary Fig. 6 Recruitment of LXRA to canonical (a) and novel (b) target gene promoters after GW3965 treatment. The promoter regions of the LXR canonical target genes *ABCA1* and *APOE* (a) and the novel target genes *TNFRSF1B*, *LCP2* and *DOK2* (b) were assessed for LXRA recruitment in macrophages 24 h post exposure to vehicle control and the LXR ligand GW3965. Peak intensity between treatments are displayed highlighting the changes in binding of LXRA within a 10 kb promoter region.
Supplementary Fig. 7 Publicly available datasets of Luminal A (25 cell lines, including MCF7) and ER-negative (25 cell lines, including MDA-MB-468) expression of the corepressors NCOR1, NCOR2/SMRT and LCOR were analysed for differential expression between BCa subtypes. CoR expression is presented as box and whisker plots with median, inter-quartile and 10-90 percentiles shown. MCF7 and MDA-MB-468 cell line expression are highlighted. Statistical significance was established using two-tailed Mann Whitney-U tests. *p<0.05.
Supplementary Fig. 8 Validation of NCOR1, NCOR2 and LCOR knock down. NCOR1 and NCOR2 in combination and LCOR alone were silenced in ER-positive LXR-luciferase cell cultures (MCF7) and ER-negative LXR-luciferase cell cultures (MDA-MB-468). Gene expression of the corepressor NCOR1 (a), NCOR2 (b) and LCOR (c) were assessed by qPCR (b) 36 h post silencing using ΔΔcT (normalised to HPRT1). Statistical analysis was established using two-way ANOVA and is representative of 2-3 independent replicates.
